# Risk Factors Influencing Survival in T‐Cell Lymphoblastic Lymphoma and T‐Cell Acute Lymphoblastic Leukemia

**DOI:** 10.1002/cam4.71365

**Published:** 2025-12-13

**Authors:** Tong Yoon Kim, Kyoung Il Min, Gi‐June Min, Ki‐Seoung Eom, Seok Lee, Seok‐Goo Cho, Seoree Kim, Jong hyuk Lee, Byung‐Su Kim, Joon won Jeoung, Hye Sung Won, Jae‐Ho Yoon, Youngwoo Jeon

**Affiliations:** ^1^ Department of Hematology, Yeoido St. Mary's Hospital, College of Medicine The Catholic University of Korea Seoul Korea; ^2^ Department of Hematology, Seoul St. Mary's Hospital, College of Medicine The Catholic University of Korea Seoul Korea; ^3^ Department of Oncology, Bucheon St. Mary's Hospital, College of Medicine The Catholic University of Korea Seoul Korea; ^4^ Department of Hematology, Incheon St. Mary's Hospital, College of Medicine The Catholic University of Korea Seoul Korea; ^5^ Department of Hematology, Eunpyeong St. Mary's Hospital, College of Medicine The Catholic University of Korea Seoul Korea; ^6^ Department of Oncology, Daejeon St. Mary's Hospital, College of Medicine The Catholic University of Korea Seoul Korea; ^7^ Department of Oncology, Uijeongbu St. Mary's Hospital, College of Medicine The Catholic University of Korea Seoul Korea

**Keywords:** allogeneic hematopoietic stem cell transplantation, autologous, T‐cell acute lymphoblastic leukemia, T‐cell lymphoblastic lymphoma

## Abstract

**Background:**

T‐cell lymphoblastic lymphoma (T‐LBL) is a rare non‐Hodgkin lymphoma. The World Health Organization defines T‐LBL and T‐cell acute lymphoblastic leukemia (T‐ALL) as the same entity. However, the clinical variations between them result in divergent treatment outcomes.

**Objectives:**

The aim of this study was to compare the outcomes of patients with T‐LBL and T‐ALL and identify ideal candidates for autologous hematopoietic stem cell transplantation (auto‐HSCT) or allogeneic hematopoietic stem cell transplantation (allo‐HSCT).

**Study Design:**

This retrospective analysis included 148 patients diagnosed with T‐LBL (67 [45.3%]) or T‐ALL (81 [54.7%]) between November 2009 and December 2022 in seven hospitals in the Republic of Korea. Overall survival (OS) and progression‐free survival (PFS) were analyzed using the Kaplan–Meier method, and allo‐HSCT, auto‐HSCT, and chemotherapy‐only treatment modalities were compared. Cox proportional hazards models were used to identify risk factors for survival, and survival decision trees were used for risk stratification.

**Results:**

The median follow‐up duration was 60 months. The 5‐year OS rates were 43.5% and 52.8% in the T‐LBL and T‐ALL groups, respectively (*p =* 0.111). The T‐LBL group had lower PFS than the T‐ALL group (*p <* 0.001). The 5‐year OS rates for allo‐HSCT, auto‐HSCT, and chemotherapy‐only were 62.8%, 62.4%, and 13%, respectively. Two or more extranodal sites, large masses > 6 cm, axial bone involvement, and non‐complete remission after chemotherapy were poor prognostic factors for OS.

**Conclusions:**

In this multicenter retrospective analysis, hematopoietic stem‐cell transplantation (allo‐ or auto‐HSCT) was associated with better survival than chemotherapy alone. For T‐LBL, an exploratory signal from our prognostic model suggests that selected high‐risk patients may be considered for upfront allo‐HSCT. However, overall survival was comparable between allo‐ and auto‐HSCT in this cohort, and durable outcomes after transplant were largely observed in patients who achieved complete remission. These findings are hypothesis‐generating and support individualized, response‐adapted strategies that warrant prospective validation.

## Graphical abstract

Survival outcomes in T‐LBL vs. T‐ALL: Implications for early allogeneic stem cell transplantation.

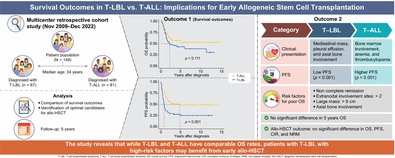

## 1 Introduction

T‐cell lymphoblastic lymphoma (T‐LBL) is a rare non‐Hodgkin lymphoma that accounts for approximately 2% of patients who undergo a neoplastic transition from immature precursor T cells [1]. The World Health Organization defines T‐LBL and T‐cell acute lymphoblastic leukemia (T‐ALL) as the same entity. T‐LBL and T‐ALL share similar genomic changes in early thymocyte subsets [2, 3]. Recent efforts have been made to distinguish T‐LBL from T‐ALL because of the different clinical features. T‐LBL typically presents with a mediastinal mass and limited bone marrow involvement (17%), whereas T‐ALL is characterized by significant bone marrow infiltration and leukemic manifestations [4, 5].

In terms of treatment, chemotherapy, similar to induction chemotherapy, was used for T‐LBL and T‐ALL [6], whereas consolidation therapies for T‐ALL differ from those of T‐LBL in the real world.

These different strategies are due to the rarity of the disease and different experiences with it, and there are questions about the possibility that T‐LBL and T‐ALL have different clinical traits. Thus, optimal candidates for allogeneic hematopoietic stem cell transplantation (allo‐HSCT) remain unclear. Patients with T‐ALL usually undergo allo‐HSCT after experiencing the first complete remission (CR) after chemotherapy [7, 8]. In contrast, for patients with T‐LBL, transplantation is considered in the second CR [9], and either autologous HSCT (auto‐HSCT) or allo‐HSCT is recommended [10, 11, 12].

Risk stratification remains a challenge. Factors like leukocytosis and early T‐cell precursor phenotype are established in T‐ALL but less clear in T‐LBL. Recent studies have identified genetic markers such as NOTCH1/FBXW7 mutations that may influence the prognosis of both entities [13].

The role of allo‐HSCT in T‐LBL, particularly as frontline therapy, remains controversial. Considering bone marrow relapses are common (39%) in T‐LBL, which has a T‐ALL‐like feature and poor outcomes after relapses, treatment concepts from T‐ALL could benefit patients with T‐LBL [4]. Identifying patients who would benefit the most from allo‐HSCT is crucial for optimizing outcomes and minimizing unnecessary toxicity.

Recent advances in targeted therapies show promise, but their integration into frontline treatment and impact on transplantation strategies are yet to be fully understood. In this study, we compared the treatment outcomes of T‐LBL and T‐ALL, focusing on factors that may guide allo‐HSCT decisions. We sought to provide insights into optimal treatment strategies and refine risk‐stratification approaches for these challenging diseases.

## 2 Materials and Methods

### 2.1 Patient Selection

We analyzed the records of 148 patients diagnosed with T‐LBL and T‐ALL between November 7, 2009, and December 15, 2022, in seven hospitals in the Republic of Korea (Yeoido, Seoul, Bucheon, Incheon, Daejeon, Eunpyeong, and Uijeongbu St. Mary's Hospital). The patients were followed up until June 2024. This multicenter retrospective cohort study included patients (a) aged ≥ 18 years, (b) who had completed a major clinical laboratory or radiology workup at the hospital, and (c) who survived for more than 1 month after diagnosis. The exclusion criteria were (a) age > 65 years, (b) HIV infection, and (c) conservative treatment, (d) patients with prior malignancies, (e) significant organ dysfunction (f) prior HSCT performed before the index hematologic diagnosis (for any other indication), and (g) patients first evaluated at our centers in relapsed/refractory status after a diagnosis elsewhere. For treatment‐modality analyses, groups were mutually exclusive; patients who later underwent allo‐HSCT after a prior auto‐HSCT were analyzed within the allo‐HSCT group. A detailed Consolidated Standards of Reporting Trials (CONSORT) Study flow diagram is presented in Figure S1.

This study adhered to the Declaration of Helsinki and was approved by the Institutional Review Board and Ethics Committee of the Catholic Medical Center in South Korea (SC23WISI0093). Due to the retrospective nature of the study, patient consent was not required.

### 2.2 Definition and Response Criteria

T‐ALL was diagnosed using a bone marrow biopsy, which revealed ≥ 20% lymphoblasts. T‐LBL is initially diagnosed by biopsy of nodal or extranodal sites and may or may not include bone marrow involvement. In the handling of borderline presentations. In this real‐world cohort, diagnostic classification followed institutional practice at the time of care. T‐ALL was defined by bone marrow lymphoblasts ≥ 20% at diagnosis, irrespective of the presence of a mediastinal mass; T‐LBL was defined by dominant nodal/extranodal disease with < 20% marrow blasts. The early T‐cell precursor (ETP) phenotype is defined as a lack of CD1a and CD8, weak CD5 expression, and positivity for one or more stem cell or myeloid markers (CD117, HLA‐DR, CD13, CD33, CD34, CD11b, or CD65) [14].

Molecular and cytogenetic data. Across participating centers, conventional karyotyping was performed on bone‐marrow aspirates; “high‐risk karyotype” was defined as including *t*(4;11), ≥ 5 abnormalities without known translocations, low hypodiploidy, MLL translocations, monosomy 7 with less than 5 abnormalities, *t*(1;19), or del(7p).

For T‐LBL, staging and response were based on the International Working Group consensus response evaluation criteria for lymphoma and the Deauville score [15]. CR was defined as the disappearance of target lesions on computed tomography with normalization of 18F‐fluorodeoxyglucose positron emission tomography‐computed tomography uptake at all sites (Deauville score, 1–3). A partial response was considered to have occurred when ≥ 30% regression was observed in the sum of the longest diameters of the target lesion. Progressive disease was defined as a > 20% increase in the tumor‐long axis or the development of a new lesion (Deauville score 5).

For T‐ALL, CR was considered when there was no evidence of leukemic blasts in the bone marrow (< 5%), complete resolution of extramedullary involvement, and recovery of the peripheral blood cell count. Relapse was regarded as the reappearance of blasts in the BM (≥ 5%) or leukemic involvement in extramedullary sites after the prior achievement of CR.

Post‐HSCT complications, including acute and chronic graft‐versus‐host disease (GVHD), were evaluated using the European Society for Blood and Marrow Transplantation scoring system [16].

### 2.3 Treatment Protocol and Conditioning Regimen

Because this was a multicenter retrospective cohort, treatment allocation and conditioning regimens were determined by treating physicians in routine practice rather than by a study protocol. For patients with T‐LBL, alternating combination chemotherapy hyper‐CVAD (fractionated cyclophosphamide, vincristine, adriamycin, and dexamethasone) and high‐dose methotrexate and cytarabine therapy were commonly used for up to six induction–consolidation chemotherapy courses [17]. Patients who achieved CR were generally considered for auto‐HSCT as consolidation therapy, whereas those who did not achieve CR (Non‐CR) or relapsed after CR were considered for allo‐HSCT.

The chemotherapy‐only group comprised patients who did not receive transplantation during follow‐up owing to patient preference, ineligibility, or disease progression. Among auto‐HSCT recipients, reduced‐intensity BuMelTT (busulfan, melphalan, thiotepa) was frequently used [18]. Among allo‐HSCT recipients, Flu–Mel–TBI (800 cGy) or ECT (TBI 1200 cGy) was commonly used [19, 20].

Patients with T‐ALL were alternately treated with modified hyper‐CVAD chemotherapy and high‐dose cytarabine‐mitoxantrone chemotherapy. At participating centers, allo‐HSCT in first CR was commonly considered. Conditioning regimens included myeloablative CY–TBI (TBI 1320 cGy) or FAT (TBI 1200 cGy), and reduced‐intensity regimens such as fludarabine–melphalan (FM) or fludarabine–busulfan (FB), as previously reported [21, 22, 23]. The choice between Reduced‐Intensity Conditioning (RIC) and myeloablative conditioning (MAC) was based on patient age, comorbidities, and disease status.

### 2.4 Statistical Analysis

Chi‐square analysis or Fisher's exact test was used to analyze categorical factors and patient characteristics, and continuous variables were compared using Student's *t* test.

Overall survival (OS) and progression‐free survival (PFS) were analyzed to determine the survival outcomes. Using the Kaplan–Meier survival curve, OS was estimated by the Kaplan–Meier method; groups were compared using the log‐rank test. PFS was defined as the proportion of patients who did not experience treatment failure, including disease progression, death, or the initiation of new chemotherapy. The risk factors affecting OS and PFS were compared using univariate and multivariate analyses with Cox proportional hazards models. We fitted multivariable Cox models for OS including treatment modality (chemotherapy‐only, auto‐HSCT, allo‐HSCT) and prespecified covariates (age > 35 years, female sex, Ann Arbor stage III–IV, extranodal sites ≥ 2, large mass > 6 cm, bone‐marrow involvement, pleural effusion, LDH elevation, WBC > 100 × 10^9^/L, high‐risk karyotype, and non‐CR after chemotherapy). Models used Efron ties. A separate model re‐referenced allo‐HSCT to obtain the auto‐HSCT versus allo‐HSCT hazard ratio. The cumulative incidence of relapse and non‐relapse mortality was analyzed by considering competing events. Gray test and fine‐gray subdistribution hazards regression were applied in univariate and multivariate analyses. Factors that were significantly (*p <* 0.05) affected by OS in the univariate analysis were entered into the multivariate analysis to determine their impact. GVHD analyses. Cumulative incidence of acute and chronic GVHD was evaluated using.

Fine–Gray subdistribution hazards with death and relapse as competing risks. Covariates included subtype (T‐ALL vs. T‐LBL), conditioning intensity (MAC vs. RIC), TBI‐based versus non‐TBI, donor type (haploidentical vs. other), and GVHD prophylaxis categorized as cyclosporine‐based versus tacrolimus‐based, according to center protocols. Estimates are reported as HRs with 95% CIs to maintain continuity with other tables.

For risk stratification, we used survival decision trees using a machine‐learning method [24]. Machine‐learning survival tree. For risk stratification, we fitted a survival decision tree using overall survival as the endpoint. Candidate predictors were prespecified and coded as binary variables aligned with our tables: age > 35 years; female sex; Ann Arbor stage III–IV; extranodal sites ≥ 2; large mass > 6 cm; bone‐marrow involvement; pleural effusion; LDH elevation; WBC > 100 × 10^9^/L; high‐risk karyotype; and post‐chemotherapy response (Non‐CR vs. CR). We specified an exponential time‐to‐event distribution and used parameter‐instability tests for splitting with *α* = 0.05. Node‐size controls were left at the package defaults (minimum split size = 40; minimum number of observations in any terminal leaf node = 20). Model size was chosen by 10‐fold cross‐validation with the 1‐SE rule to favor parsimony. Stability was examined via bootstrap refitting, summarizing split‐selection frequencies and risk‐group assignment consistency.

Statistical significance was defined as *p <* 0.05. The calculated *p* values were two‐sided. All calculations were performed using R software version 4.0.2 (R Foundation for Statistical Computing, Vienna, Austria).

## 3 Results

### 3.1 Patients' Characteristics

This study analyzed a total of 148 patients, comprising 67 (45.3%) with T‐LBL and 81 (54.7%) with T‐ALL (Table 1). The median age of the entire cohort was 34 years (range 18–65 years), with a male predominance (62.8%) observed in both groups. There were significantly more cases of extranodal sites ≥ 2, large mass > 6 cm, pleural effusion, mediastinal involvement, axial bone involvement, and CD3 positivity in the T‐LBL group than in the T‐ALL group. Additionally, there were significantly more cases of bone marrow involvement, hepatic involvement, leukocytosis > 100 × 10^9^/L, hemoglobin level < 12 g/dL, platelet count < 100 × 10^9^/L, LDH elevation above the normal range, CD34 positivity, and allo‐HSCT in the T‐ALL group than in the T‐LBL group. Baseline molecular/cytogenetic features and outcomes. The high‐risk karyotype group was observed in 27 (18.2%) overall and did not differ significantly between T‐ALL and T‐LBL (*p* = 0.111; Table 1).

**TABLE 1:** Patient characteristics.

Variable	Total	T‐ALL, *N* = 81	T‐LBL, *N* = 67	*p*
Demographics				
Sex				> 0.999
Female, *N* (%)	40 (27.0)	22 (27.2)	18 (26.9)	
Male, *N* (%)	108 (73.0)	59 (72.8)	49 (73.1)	
Age at diagnosis > 35 years, *N* (%)	52 (35.1)	31 (38.3)	21 (31.3)	0.48
Disease burden/sites				
Extranodal sites ≥ 2, *N* (%)^a^	85 (57.4)	35 (43.2)	50 (74.6)	< 0.001
Large mass > 6 cm, *N* (%)	37 (25.0)	10 (12.3)	27 (40.3)	< 0.001
Bone marrow involvement, *N* (%)	120 (81.1)	81 (100.0)	39 (58.2)	< 0.001
Pleural effusion, *N* (%)	39 (26.4)	7 (8.6)	32 (47.8)	< 0.001
Hepatomegaly, *N* (%)	17 (11.5)	14 (17.3)	3 (4.5)	0.03
Splenomegaly, *N* (%)	33 (22.3)	23 (28.4)	10 (14.9)	0.078
Mediastinal involvement, *N* (%)	83 (56.1)	29 (35.8)	54 (80.6)	< 0.001
Pericardial involvement, *N* (%)	19 (12.8)	7 (8.6)	12 (17.9)	0.152
Axial bone involvement, *N* (%)	20 (13.5)	4 (4.9)	16 (23.9)	0.002
Laboratory				
WBC count > 100 × 10^9^/L, *N* (%)	20 (13.5)	20 (24.7)	0 (0.0)	< 0.001
Hemoglobin level < 12 g/dL, *N* (%)	80 (54.1)	57 (70.4)	23 (34.3)	< 0.001
Platelet count < 100 × 10^9^/L, *N* (%)	58 (39.2)	46 (56.8)	12 (17.9)	< 0.001
LDH elevation, *N* (%)	98 (66.2)	63 (77.8)	35 (52.2)	0.002
Immunophenotype/Genetics				
CD3 positive, *N* (%)	118 (84.3)	57 (75.0)	61 (95.3)	0.002
CD5 positive, *N* (%)	95 (76.6)	60 (80.0)	35 (71.4)	0.376
CD10 positive, *N* (%)	54 (42.9)	33 (45.2)	21 (39.6)	0.658
CD34 positive, *N* (%)	55 (46.6)	43 (58.9)	12 (26.7)	0.001
High‐risk karyotype group, *N* (%)^b^	27 (18.2)	19 (23.5)	8 (11.9)	0.111
Treatment exposure				
Non‐CR, *N* (%)	33 (22.3)	10 (12.3)	23 (34.3)	0.003
Auto‐HSCT, *N* (%)^c^	17 (11.5)	0 (0.0)	17 (25.4)	< 0.001
Allo‐HSCT, *N* (%)	89 (60.1)	63 (77.8)	26 (38.8)	< 0.001
Years from diagnosis to transplantation, mean (25%, 75% quantile)	0.5 (0.5, 0.7)	0.5 (0.4, 0.6)	0.7 (0.6, 0.8)	< 0.001

Abbreviations: allo‐HSCT, allogeneic hematopoietic stem cell transplantation; auto‐HSCT, autologous hematopoietic stem cell transplantation; LDH, Lactate dehydrogenase; *N*, number; Non‐CR, other than complete remission; T‐ALL, T‐cell acute lymphoblastic leukemia; T‐LBL, T‐cell lymphoblastic lymphoma; WBC, white blood cell.

^a^ Extranodal sites are defined as organ involvement other than nodal sites. These include pleural effusion but exclude splenomegaly or hepatomegaly.

^b^ High risk group included *t*(4;11), ≥ 5 abnormalities without known translocations, low hypodiploidy, MLL translocations, monosomy 7 with less than 5 abnormalities, *t*(1;19), or del(7p).

^c^ Auto‐HSCT counts patients who did not subsequently undergo allo‐HSCT (i.e., auto‐only). Allo‐HSCT includes any patient who received allo‐HSCT at any time during the study period, including three patients who underwent allo‐HSCT after a prior auto‐HSCT.

Of 148 patients, 89 (60.1%) underwent allo‐HSCT, 17 (11.5%) auto‐HSCT, and 42 (28.4%) received chemotherapy only (Table 1). By subtype, allo‐HSCT was performed in 63 (77.8%) of T‐ALL and 26 (38.8%) of T‐LBL; auto‐HSCT was used predominantly in T‐LBL (17, 25.4%) and not in T‐ALL. Time from diagnosis to HSCT was 0.5 years in T‐ALL versus 0.7 years in T‐LBL (*p* < 0.001; Table 1). Among the allo‐HSCT group (*n* = 89), conditioning was MAC in 52 (58.4%) and RIC in 37 (41.6%); TBI‐based regimens were used in 74 (83.1%) overall. MAC use was substantially higher in T‐ALL (79.4%) than in T‐LBL (7.7%) (*p* < 0.001; Table S1).

### 3.2 Patient Survival Outcome and Risk Factors

**FIGURE 1:**
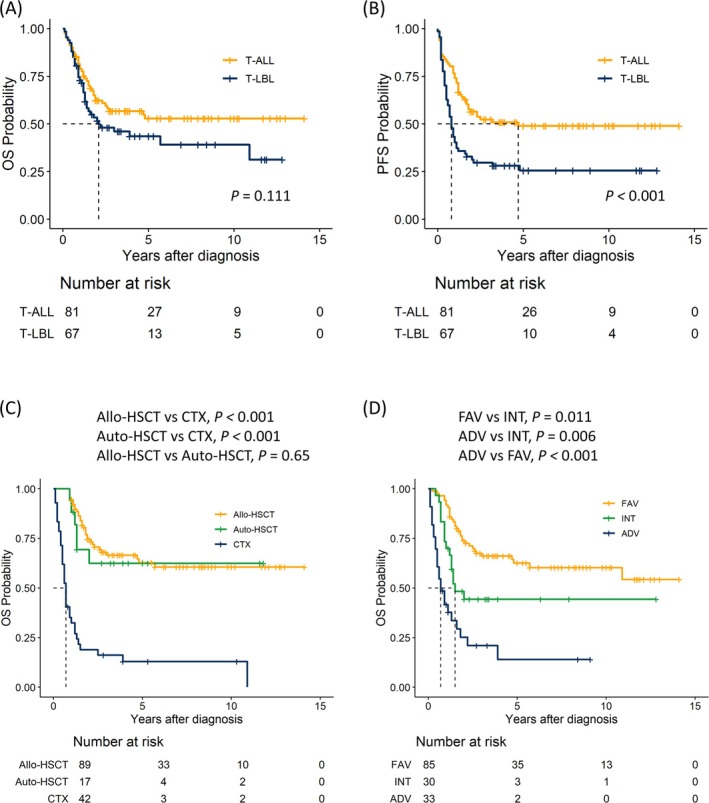
Survival outcomes for patients with T‐LBL and T‐ALL. (A) OS by subtype, (B) PFS by subtype, (C) OS by allo‐HSCT, auto‐HSCT, and CTX. (D) OS by risk stratification group according to the survival tree algorithm. ADV, adverse group; allo‐HSCT, allogeneic hematopoietic stem cell transplantation; auto‐HSCT, autologous hematopoietic stem cell transplantation; CTX, chemotherapy; FAV, favorable group; INT, intermediate group; OS, overall survival; PFS, progression‐free survival; T‐ALL, T‐cell acute lymphoblastic leukemia; T‐LBL, T‐lymphoblastic lymphoma.

**FIGURE 2:**
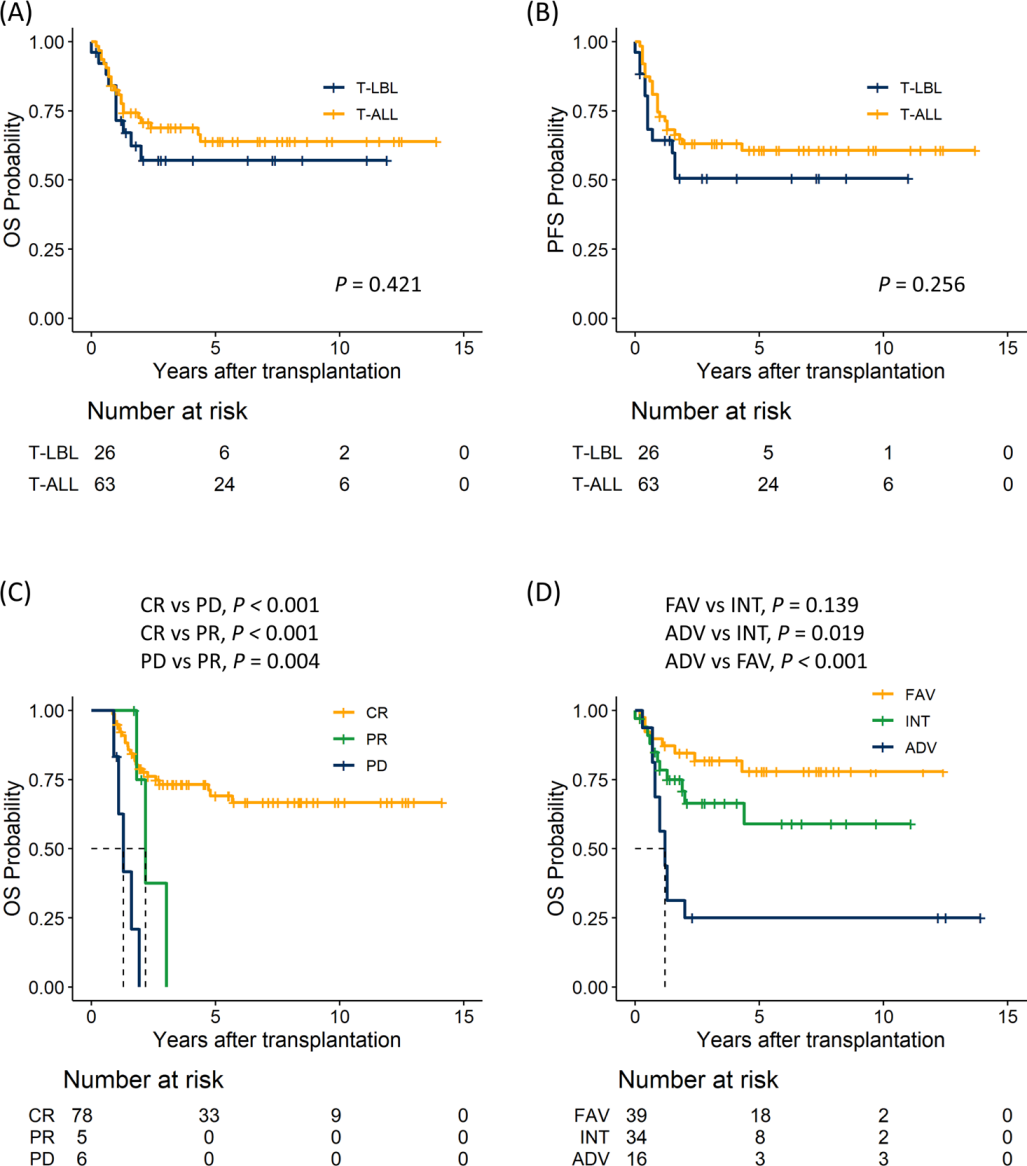
Survival outcomes of patients who underwent allogeneic hematopoietic stem cell transplantation. (A) OS by subtype, (B) PFS by subtype, and (C) OS according to pre‐transplantation disease status. (D) OS in the risk‐stratified group. CR, complete remission; OS, overall survival; PD, progressive disease; PFS, progression‐free survival; PR, partial response.

In 148 patients, the median follow‐up period was 60 (10–170) months for survivors, and the median OS was 4.7 years (95% confidence interval [CI]: 3.8–5.6 years). The 5‐year OS rates in the T‐LBL and T‐ALL groups were 43.5% (95% CI, 32.4%–58.4%) and 52.8% (95% CI, 42.5%–65.7%), respectively. There were no differences in OS between the two groups (*p =* 0.111), whereas PFS was lower in the T‐LBL group (*p <* 0.001) (Figures 1A and 2B).

The 5‐year OS rates in the allo‐HSCT, auto‐HSCT, and chemotherapy‐only (CTX) groups were 62.8% (52.8%–74.7%), 62.4% (42.5%–91.6%), and 13% (5.6%–30.1%), respectively. Allo‐ and auto‐HSCT showed comparable overall survival, whereas both were superior to chemotherapy alone in this retrospective cohort (Figure 1C). In the adjusted Cox model, both transplant modalities showed lower mortality risk versus chemotherapy alone. However, the allo‐HSCT versus auto‐HSCT comparison remained inconclusive given wide confidence intervals (Table S2). Estimates were directionally consistent with the unadjusted Kaplan–Meier curves.

For risk stratification, we used a decision survival tree algorithm for machine learning. The algorithm split the Non‐CR group into an adverse group. In the CR group, a large mass (> 6 cm) was included in the intermediate group. Patients with CR status without a large lymphoma mass comprised the favorable group. The 5‐year OS rates in the favorable, intermediate, and adverse groups were 62.5% (52.6%–74.3%), 44.3% (29.2%–67.2%), and 14% (4.7%–41.6%), respectively (Figure 1D).

In the multivariate analysis of OS, a large mass > 6 cm, axial bone involvement, and response after chemotherapy other than CR were risk factors that significantly affected the outcome (Table 2). Extranodal sites ≥ 2 and Non‐CR were factors associated with shorter PFS. Cumulative incidence of relapse was associated with large lymphomas (> 6 cm). This finding showed that chemotherapy resistance was associated with extranodal sites ≥ 2, whereas a large size affected the probability of relapse after CR (Table S3). Together, these factors were associated with poorer overall survival. In addition, the subgroup analysis suggested that the benefit of allogeneic HSCT was most pronounced in the intermediate‐ and high‐risk groups. In contrast, patients in the favorable risk group had outcomes similar to those of chemotherapy alone or auto‐HSCT.

**TABLE 2:** Univariate and multivariate analyses of the overall survival outcomes.

Variable	Univariate		Multivariate	
	HR, 95% CI	*p*	HR, 95% CI	*p*
Age at diagnosis > 35 versus 35 years	1.3 (0.81, 2.08)	0.278		
Female versus male	1.56 (0.96, 2.52)	0.074		
Ann Arbor stage III–IV versus I–II	1.74 (0.55, 5.53)	0.348		
Extranodal sites ≥ 2 versus < 2	1.8 (1.1, 2.95)	0.019	1.11 (0.62, 2)	0.727
Large mass > 6 cm versus ≤ 6 cm	1.75 (1.05, 2.9)	0.031	1.78 (1.01, 3.14)	0.046
Bone marrow involvement versus none	1.02 (0.56, 1.86)	0.952		
Pleural effusion versus none	1.06 (0.63, 1.8)	0.817		
Hepatic involvement versus none	0.52 (0.21, 1.29)	0.161		
Splenic involvement versus none	0.89 (0.5, 1.6)	0.701		
Mediastinal involvement versus none	1.37 (0.85, 2.19)	0.196		
Pericardial involvement versus none	1.74 (0.97, 3.11)	0.065		
Axial bone involvement versus none	2.24 (1.26, 3.97)	0.006	2.34 (1.25, 4.4)	0.008
WBC counts > 100 versus ≤ 100 × 10^9^/L	1.13 (0.58, 2.2)	0.723		
Hemoglobin level < 12 versus ≥ 12 g/dL	1.18 (0.74, 1.86)	0.491		
Platelet count < 100 versus ≥ 100 × 10^9^/L	1.1 (0.69, 1.76)	0.686		
LDH elevation versus normal	1.35 (0.81, 2.25)	0.244		
High‐risk karyotype versus the other	1.49 (0.86, 2.57)	0.151		
ETP versus non‐ETP	0.91 (0.5, 1.65)	0.749		
Non‐CR versus CR	4.25 (2.59, 6.98)	< 0.001	4.59 (2.76, 7.64)	< 0.001
T‐LBL versus T‐ALL	1.45 (0.92, 2.3)	0.112		

Abbreviations: CI, confidence interval; ETP, early T‐cell precursor; HR, hazard ratio; LDH, lactate dehydrogenase; NA, not applicable; Non‐CR, other than complete remission; T‐ALL, T‐cell acute lymphoblastic leukemia; T‐LBL, T‐cell lymphoblastic lymphoma; WBC, white blood cell.

### 3.3 Patient Allogeneic Hematopoietic Stem Cell Transplantation Outcomes

Patients who underwent allo‐HSCT showed no differences between T‐LBL and T‐ALL in terms of OS, PFS, cumulative incidence of relapse, or cumulative incidence of non‐relapse mortality (Figures 2A,B and S2A,B). Regarding the pre‐transplantation response of all patients, 78 experienced CR, five experienced partial response, and six experienced progressive disease (detailed patient characteristics are shown in Table S1). Each response group was significantly different in terms of survival; however, only the CR group showed long‐term survival (Figure 2C). In the risk stratification by survival tree, the intermediate and favorable groups showed no differences in OS outcomes (Figure 2D). This pattern is consistent with durable outcomes after HSCT among CR achievers, irrespective of donor source, but our data are underpowered to infer modality superiority. Because the CTX arm included patients unable to proceed to HSCT (by design of real‐world care), transplant versus CTX differences likely reflect selection by response/eligibility as well as treatment effect. The incidence of acute GVHD and chronic GVHD was higher in the T‐ALL group than in the T‐LBL group, although the severe form of the GVHD was similar in both groups (Figure S2C–F). More patients in the T‐ALL group than in the T‐LBL group underwent myeloablative chemotherapy regimens, which may partly explain the higher incidence of acute GVHD in the former versus latter group (Tables S1 and S3). In competing‐risks models, MAC was an independent predictor of acute GVHD (HR 2.88, 95% CI 1.06–7.82; *p* = 0.038), whereas donor type (haploidentical vs. other) and prophylaxis (cyclosporine‐based vs. tacrolimus‐based) were not significant for either acute or chronic GVHD. The univariable excess of acute/chronic GVHD in T‐ALL versus T‐LBL attenuated after adjusting for MAC and other covariates (Table S4).

## 4 Discussion

This study compared T‐ALL and T‐LBL treatment outcomes to determine a better strategy for patients eligible for HSCT. The clinical presentation of each group was different. T‐LBL mainly presented as a mass effect, whereas T‐ALL showed leukocytosis, anemia, and thrombocytopenia. A major finding of our study was the comparable OS between patients with T‐LBL and T‐ALL (5‐year OS: 43.5% vs. 52.8%, *p* = 0.111), despite significant differences in initial disease characteristics and treatment strategies. This finding aligns with recent studies suggesting a biological continuum between T‐LBL and T‐ALL. However, the inferior PFS observed in patients with T‐LBL (*p <* 0.001) underscores the need for refined treatment approaches in this subgroup.

Our data showed that different treatment approaches were used for the two diseases at varying times, from diagnosis to treatment. Many patients with T‐LBL made an effort to complete the six cycles of chemotherapy, whereas patients with T‐ALL proceeded with allo‐HSCT after induction and consolidation chemotherapy.

These real‐world strategy differences are based on the finding that in patients with T‐LBL, pediatric‐like ALL therapy with high‐dose chemotherapy could achieve long‐term survival, with a 3‐year OS of 69.2% and a 7‐year OS of 51%. However, these studies reported relapse rates of 26%–36% [25, 26]. Furthermore, the auto‐HSCT strategy can prolong OS by 79.1% and 3‐year OS by 60% for 5‐year OS [10, 11]. These published outcomes were similar to those of our study (62.4%). Overall, the rarity of the disease and good experiences with patients who responded to chemotherapy resulted in various strategies being adopted among the centers.

Risk stratification in previous studies was inconclusive, except for responses to chemotherapy [13]. We hypothesize that the separate analysis for T‐ALL and T‐LBL caused this discrepancy. For example, leukocytosis and ETP subtypes, which are adverse factors in T‐ALL, were not present even in refractory T‐LBL [7, 8]. Moreover, Yoon et al. showed similar survival outcomes among patients with and without ETP, even with a difference in genomic traits between the two groups [27]. Hu et al. reported Non‐CR response, LDH elevation, and pleural effusion as risk factors, indicating that different proportions of the T‐LBL and T‐ALL cohorts had different prognostic factors [11].

An analysis of T‐LBL and T‐ALL is needed because overlapping clinical presentations were observed in the two groups: T‐LBL with bone marrow involvement and T‐ALL with extramedullary involvement. This overlap of clinical features makes it difficult to stratify the risk using the Cox regression hazard ratio scoring system. The survival decision tree method was used to improve risk stratification. This method is used for subgrouping and stratification and can compare many decision trees.

In the risk stratification, we found that the size of the lymphoma mass could contribute to relapse after CR. Patients at high risk for relapse have a chance for transplantation, whereas patients with a refractory status often fail to receive consolidative treatment. In this study, there were no differences in the OS between the T‐LBL and T‐ALL groups. However, PFS was inferior in the T‐LBL group compared with the T‐ALL group. By not choosing the early application of the allo‐HSCT, which was the routine protocol in the T‐ALL, relapse cases could be higher in the T‐LBL group than in T‐ALL. In our study, three patients underwent allo‐HSCT after auto‐HSCT; these patients were all extranodal and involved more than two sites. Of the 37 patients in the large‐mass group, 12 (32.4%) failed to undergo transplantation and died of disease progression. Early application of allo‐HSCT was effective in patients with ALL, such as Philadelphia chromosome‐like ALL, which is reported to have a high incidence of relapse based on a chemotherapy maintenance strategy [28, 29].

Allo‐HSCT outcomes did not differ between the two groups. In the T‐LBL, patients underwent a RIC rather than T‐ALL. Caution is required to conclude that the RIC is preferred over MAC because most RIC conditionings were TBI‐based in this study. TBI‐based conditionings are usually developed to intensify concurrent chemotherapy with RIC. HSCT outcomes are largely related to the pretransplantation disease status, which means that further efforts are required to clear the tumor. In addition to hyper‐CVAD protocols, new combination therapies could be considered, such as nelarabine‐ and venetoclax‐based therapy [30].

The higher incidence of acute GVHD observed in patients with T‐ALL is an intriguing finding that warrants further investigation. However, this did not translate into significant differences in survival outcomes and their severity.

Our study has limitations in terms of the number of patients, but it could be the most extensive study of its kind because it included centers with the same treatment strategy. Second, there was a lack of genomic data. Genomic limitations under the WHO 5th framework. While the WHO 5th edition emphasizes integrated morphologic, immunophenotypic, and genetic criteria for lymphoid neoplasms, our real‐world, multicenter dataset lacked uniform targeted sequencing (e.g., *NOTCH1/FBXW7, PTEN, DNMT3A*) and thus could not compare molecular landscapes between T‐LBL and T‐ALL. Prospective studies with standardized molecular profiling are needed to validate. We evaluated bone marrow aspirates and performed conventional karyotyping. However, bone marrow involvement was less predominant in the T‐LBL group than in the T‐ALL group, and we encountered problems that needed clustering of the small number of traits, consistent with a previous study. For this reason, we only compared previous adverse karyotypes and focused on the clinical characteristics [27, 31]. Third, the ETP diagnosis was made by the pathologists of each center; therefore, the diagnosis might have been affected by the number of biopsy specimens and facilities. Fourth, subgroup analyses—particularly auto‐HSCT—were underpowered, yielding wide CIs even after covariate adjustment; accordingly, we avoid strong comparative claims between allo‐ and auto‐HSCT. These subgroup findings should be considered hypothesis‐generating and require prospective validation. Finally, owing to its retrospective nature, the results must be validated in a prospective study. However, this study pointed out the real‐world strategy for T‐LBL in various centers and attempted to draw attention to the T‐ALL strategy.

In conclusion, transplantation—allo or auto—was associated with improved survival versus chemotherapy alone, but we did not demonstrate a consistent overall survival advantage of allo‐ over auto‐HSCT. In T‐LBL, high‐risk features highlighted by our model (non‐CR and bulky disease) may justify consideration of early allo‐HSCT in selected patients, particularly when complete remission can be achieved before transplant; nevertheless, this strategy remains exploratory given the retrospective design and the small auto‐HSCT subgroup. We therefore favor an individualized, response‐adapted approach and encourage prospective studies to validate these observations.

## Author Contributions

**Tong Yoon Kim:** writing – original draft, data curation, formal analysis. **Kyoung Il Min:** data curation, writing – review and editing. **Gi‐June Min:** data curation, writing – review and editing. **Ki‐Seoung Eom:** data curation, writing – review and editing. **Seok Lee:** data curation, writing – review and editing. **Seok‐Goo Cho:** data curation, writing – review and editing. **Seoree Kim:** resources, writing – review and editing. **Jong hyuk Lee:** resources, writing – review and editing. **Byung‐Su Kim:** writing – review and editing, resources. **Joon won Jeoung:** validation, writing – review and editing. **Hye Sung Won:** validation, writing – review and editing. **Jae‐Ho Yoon:** conceptualization, supervision, data curation, writing – review and editing, formal analysis. **Youngwoo Jeon:** conceptualization, data curation, supervision, writing – review and editing, formal analysis.

## Ethics Statement

This study adhered to the Declaration of Helsinki and was approved by the Institutional Review Board and Ethics Committee of the Catholic Medical Center in South Korea (SC23WISI0093).

## Consent

Due to the retrospective nature of the study, patient consent was not required.

## Conflicts of Interest

The authors declare no conflicts of interest.

## Supporting information


**Table S1:** Characteristic of patients with allogeneic hematopoietic stem cell transplantation.
**Table S2:** Adjusted Cox model for overall survival.
**Table S3:** Univariate and multivariate analyses of the Progression‐free survival and Cumulative incidence of relapse.
**Table S4:** Univariate and multivariate analyses of the GVHD survival outcomes of patients with allogeneic hematopoietic stem cell transplantation.
**Figure S1:** Study flow diagram.
**Figure S2:** Transplantation outcomes for patients with T‐LBL and T‐ALL. (A) Cumulative incidence of relapse by subtype, (B) cumulative incidence of NRM by subtype, (C) cumulative incidence of acute GVHD by subtype, (D) cumulative incidence of grade III or IV acute GVHD by subtype, (E) cumulative incidence of chronic GVHD by subtype, (F) cumulative incidence of moderate or severe chronic GVHD by subtype.

## Data Availability

The data that support the findings of this study are available on request from the corresponding author. The data are not publicly available due to privacy or ethical restrictions.
