# Supplementary material for: Risk Factors Influencing Survival in T‐Cell Lymphoblastic Lymphoma and T‐Cell Acute Lymphoblastic Leukemia
Source: Cancer Med. 2025 Dec 13;14(24):e71365. doi: 10.1002/cam4.71365 (PMC12701560; doi:10.1002/cam4.71365)
Supplement: Supplementary file 1 — Table S1: Characteristic of patients with allogeneic hematopoietic stem cell transplantation. Table S2: Adjusted Cox model for overall survival. Table S3: Univariate and multivariate analyses of the Progression‐free survival and Cumulative incidence of relapse. Table S4: Univariate and multivariate analyses of the GVHD survival outcomes of patients with allogeneic hematopoietic stem cell transplantation. Figure S1: Study flow diagram. Figure S2: Transplantation outcomes for patients with T‐LBL and T‐ALL. (A) Cumulative incidence of relapse by subtype, (B) cumulative incidence of NRM by subtype, (C) cumulative incidence of acute GVHD by subtype, (D) cumulative incidence of grade III or IV acute GVHD by subtype, (E) cumulative incidence of chronic GVHD by subtype, (F) cumulative incidence of moderate or severe chronic GVHD by subtype. [file CAM4-14-e71365-s001.docx]

**Supplementary material**

**Title:** Risk factors influencing survival in T-cell lymphoblastic lymphoma and T-cell acute lymphoblastic leukemia: Implications for allogeneic transplantation strategies

Tong Yoon Kim, Kyoung Il Min, Gi-June Min, Ki-Seoung Eom, Seok Lee, Seok-Goo Cho, Seoree Kim, Jong hyuk Lee, Byung-Su Kim, Joon won Jeoung, Hye Sung Won, Jae-Ho Yoon, Youngwoo Jeon

**Table S1.** Characteristic of patients with allogeneic hematopoietic stem cell transplantation

| Variable | Total, N=89 | T-ALL, N=63 | T-LBL, N=26 | *P* value |
| --- | --- | --- | --- | --- |
| Sex |  |  |  | 0.788 |
| Female, N (%) | 24 (27.0) | 18 (28.6) | 6 (23.1) |  |
| Male, N (%) | 65 (73.0) | 45 (71.4) | 20 (76.9) |  |
| Age at diagnosis >35 years, N (%) | 30 (33.7) | 21 (33.3) | 9 (34.6) | >0.999 |
| Extranodal sites ≥2, N (%) | 46 (51.7) | 26 (41.3) | 20 (76.9) | 0.005 |
| **Large** mass >6 cm, N (%) | 13 (14.6) | 5 (7.9) | 8 (30.8) | 0.015 |
| High-risk karyotype group, N (%) | 16 (18.0) | 13 (20.6) | 3 (11.5) | 0.476 |
| Pre-transplantation status |  |  |  | <0.001 |
| CR1, N (%) | 78 (79.8) | 62 (98.4) | 16 (61.5) |  |
| >CR, N (%) | 7 (7.9) | 4 (6.3) | 3 (11.5) |  |
| Partial remission, N (%) | 5 (5.6) | 0 (0) | 5 (19.2) |  |
| Progressive disease, N (%) | 6 (6.7) | 1 (1.6) | 5 (19.2) |  |
| Donor type |  |  |  | 0.032 |
| Matched sibling donor | 23 (25.8) | 16 (25.4) | 7 (26.9) |  |
| Unrelated donor | 45 (50.6) | 33 (52.4) | 12 (46.2) |  |
| Double unit cord blood | 12 (13.5) | 11 (17.5) | 1 (3.8) |  |
| Haploidentical donor | 9 (10.1) | 33 (52.4) | 12 (46.2) |  |
| TBI-based conditioning, N (%) | 74 (83.1) | 51 (81.0) | 23 (88.5) | 0.583 |
| MAC conditioning, N (%) | 52 (58.4) | 50 (79.4) | 2 (7.7) | <0.001 |

N, number; T-LBL, T-cell lymphoblastic lymphoma; T-ALL, T-cell acute lymphoblastic leukemia; CR1, complete remission; >CR, complete remission after receiving salvage chemotherapy for a first or more relapse; TBI, total body irradiation; MAC, myeloablative conditioning.

**Table S2.** Adjusted Cox model for overall survival

| Variable | HR, 95% CI | *P* |
| --- | --- | --- |
| Auto-HSCT vs CTX-only | 0.13 (0.04, 0.39) | <0.001 |
| Allo-HSCT vs CTX-only | 0.14 (0.08, 0.27) | <0.001 |
| Auto-HSCT vs Allo-HSCT | 0.95 (0.31, 2.6) | 0.846 |
| Age at diagnosis >35 vs. 35 years | 0.95 (0.54, 1.67) | 0.853 |
| Female vs. male | 2.27 (1.32, 3.9) | 0.003 |
| Ann Arbor stage III-IV vs. I-II | 0.98 (0.26, 3.71) | 0.976 |
| Extranodal sites ≥2 vs. <2 | 1.01 (0.52, 1.93) | 0.989 |
| Large mass >6 cm vs. ≤6 cm | 1.62 (0.84, 3.12) | 0.15 |
| Bone marrow involvement vs. none | 0.85 (0.38, 1.87) | 0.677 |
| Pleural effusion vs. none | 0.67 (0.34, 1.33) | 0.251 |
| LDH elevation vs. normal | 1.11 (0.62, 2) | 0.731 |
| WBC counts >100 vs. ≤100 × 10^9^/L | 1.93 (0.91, 4.09) | 0.088 |
| High-risk karyotype vs. the other | 1.62 (0.99, 2.64) | 0.055 |
| Non-CR vs. CR | 5.13 (2.7, 9.74) | <0.001 |

Auto-HSCT, autologous hematopoietic hematopoietic stem cell transplantation; Allo-HSCT, allogeneic hematopoietic stem cell transplantation; HR, hazard ratio; CI, confidence interval; *P*, *P* value; LDH, lactate dehydrogenase; WBC, white blood cell; ETP, early T-cell precursor; Non-CR, other than complete remission.

**Table S3.** Univariate and multivariate analyses of the Progression-free survival and Cumulative incidence of relapse

|  | Univariate | | Multivariate | |
| --- | --- | --- | --- | --- |
| Variable | HR, 95% CI | *P* | HR, 95% CI | *P* |
| **Progression-free survival** | | | | |
| Age at diagnosis >35 vs. 35 years | 1.05 (0.68, 1.62) | 0.825 |  |  |
| Female vs. male | 1.18 (0.75, 1.85) | 0.478 |  |  |
| Ann Arbor stage III-IV vs. I-II | 1.5 (0.55, 4.1) | 0.426 |  |  |
| Extranodal sites ≥2 vs. <2 | 2.11 (1.35, 3.31) | 0.001 | 1.89 (1.13, 3.18) | 0.016 |
| **Large** mass >6 cm vs. ≤6 cm | 1.85 (1.17, 2.9) | 0.008 | 1.61 (0.96, 2.72) | 0.073 |
| Bone marrow involvement vs. none | 0.85 (0.51, 1.43) | 0.549 |  |  |
| Pleural effusion vs. none | 1.46 (0.93, 2.3) | 0.101 |  |  |
| Hepatic involvement vs. none | 0.48 (0.21, 1.09) | 0.079 |  |  |
| Splenic involvement vs. none | 0.88 (0.53, 1.48) | 0.635 |  |  |
| Mediastinal involvement vs. none | 1.78 (1.15, 2.76) | 0.009 |  |  |
| Pericardial involvement vs. none | 1.86 (1.08, 3.21) | 0.025 |  |  |
| Axial bone involvement vs. none | 1.85 (1.09, 3.15) | 0.023 |  |  |
| WBC count >100 vs. ≤100 × 10^9^/L | 0.84 (0.45, 1.58) | 0.594 |  |  |
| Hemoglobin level <12 vs. ≥12 g/dL | 0.94 (0.62, 1.43) | 0.781 |  |  |
| Platelet count <100 vs. ≥100 × 10^9^/L | 0.93 (0.6, 1.42) | 0.731 |  |  |
| LDH elevation vs. normal | 1.24 (0.79, 1.95) | 0.344 |  |  |
| High-risk karyotype vs. the other | 1.3 (0.78, 2.16) | 0.308 |  |  |
| ETP vs. non-ETP | 0.44 (0.21, 0.96) | 0.04 | 0.59 (0.26, 1.36) | 0.219 |
| Non-CR vs. CR | 8.95 (5.63, 14.2) | <0.001 | 9.24 (5.56, 15.4) | <0.001 |
| T-LBL vs. T-ALL | 2.06 (1.35, 3.13) | <0.001 | 0.93 (0.55, 1.56) | 0.77 |
| **Cumulative incidence of relapse** | | | | |
| Age at diagnosis >35 vs. 35 years | 0.93 (0.47, 1.84) | 0.830 |  |  |
| Female vs. male | 1.00 (0.49, 2.02) | >0.999 |  |  |
| Ann Arbor stage III-IV vs. I-II | 2.41 (0.33, 17.6) | 0.390 |  |  |
| Extranodal sites ≥2 vs. <2 | 2.55 (1.22, 5.33) | 0.013 | 2.23 (0.84, 5.93) | 0.110 |
| **Large** mass >6 cm vs. ≤6 cm | 4.52 (2.36, 8.64) | <0.001 | 3.29 (1.21, 8.91) | 0.020 |
| Bone marrow involvement vs. none | 0.54 (0.26, 1.12) | 0.098 |  |  |
| Pleural effusion vs. none | 2.27 (1.18, 4.37) | 0.014 | 1.04 (0.48, 2.25) | 0.930 |
| Hepatic involvement vs. none | 0.71 (0.21, 2.37) | 0.570 |  |  |
| Splenic involvement vs. none | 0.29 (0.09, 0.97) | 0.045 | 0.36 (0.09, 1.42) | 0.140 |
| Mediastinal involvement vs. none | 2.32 (1.14, 4.72) | 0.020 | 0.57 (0.23, 1.37) | 0.210 |
| Pericardial involvement vs. none | 1.35 (0.59, 3.09) | 0.480 |  |  |
| Axial bone involvement vs. none | 1.71 (0.76, 3.86) | 0.200 |  |  |
| WBC count >100 vs. ≤100 × 10^9^/L | 0.82 (0.29, 2.3) | 0.700 |  |  |
| Hemoglobin level <12 vs. ≥12 g/dL | 0.84 (0.44, 1.6) | 0.600 |  |  |
| Platelet count <100 vs. ≥100 × 10^9^/L | 0.89 (0.45, 1.74) | 0.730 |  |  |
| LDH elevation vs. normal | 1.33 (0.64, 2.75) | 0.440 |  |  |
| High-risk karyotype vs. the other | 0.67 (0.27, 1.67) | 0.390 |  |  |
| ETP vs. non-ETP | †NA (NA, NA) |  |  |  |
| Non-CR vs. CR | †NA (NA, NA) |  |  |  |
| T-LBL vs. T-ALL | 2.07 (1.07, 4.02) | 0.031 | 1.19 (0.55, 2.55) | 0.660 |

HR, hazard ratio; CI, confidence interval; *P,* *P* value; NA, not applicable; WBC, white blood cell; LDH, lactate dehydrogenase; ETP, early T-cell precursor; Non-CR, other than complete remission; T-LBL, T-cell lymphoblastic lymphoma; T-ALL, T-cell acute lymphoblastic leukemia. †NA (not estimable): All patients in this stratum died without prior relapse, resulting in zero relapse events and precluding estimation of the cumulative incidence of relapse (Fine–Gray).

**Table S4.** Univariate and multivariate analyses of the GVHD survival outcomes of patients with allogeneic hematopoietic stem cell transplantation

|  | Univariate | | Multivariate | |
| --- | --- | --- | --- | --- |
|  | HR, 95% CI | *P* | HR, 95% CI | *P* |
| Cumulative incidence of acute GVHD | | | | |
| T-ALL vs T-LBL | 5.44 (1.61, 18.4) | 0.006 | 0.39 (0.09, 1.65) | 0.2 |
| MAC vs RIC conditioning | 4.62 (1.97, 10.82) | <0.001 | 2.88 (1.06, 7.82) | 0.038 |
| TBI-based vs non-TBI conditioning | 1.41 (0.59, 3.39) | 0.44 |  |  |
| Cyclosporine-based vs. Tacrolimus-based protocols | 1.01 (0.5, 2.06) | 0.98 |  |  |
| Haploidentical Donor vs the other | 0.43 (0.12, 1.54) | 0.19 |  |  |
| Cumulative incidence of chronic GVHD | | | | |
| T-ALL vs T-LBL | 3.55 (1.26, 10.1) | 0.017 |  |  |
| MAC vs RIC conditioning | 1.44 (0.73, 2.81) | 0.29 |  |  |
| TBI based vs non-TBI conditioning | 0.55 (0.28, 1.08) | 0.082 |  |  |
| Cyclosporine-based vs. Tacrolimus-based-protocols | 1.28 (0.64, 2.58) | 0.49 |  |  |
| Haploidentical Donor vs the other | 0.24 (0.03, 1.88) | 0.17 |  |  |

GVHD, graft versus host disease; HR, hazard ratio; CI, confidence interval; *P*, *P* value; T-LBL, T-cell lymphoblastic lymphoma; T-ALL, T-cell acute lymphoblastic leukemia; MAC, myeloablative conditioning; RIC, reduced-intensity conditioning; TBI, total body irradiation.

**Figure S1.** Study flow diagram.

T-LBL, T-lymphoblastic lymphoma; T-ALL, T-
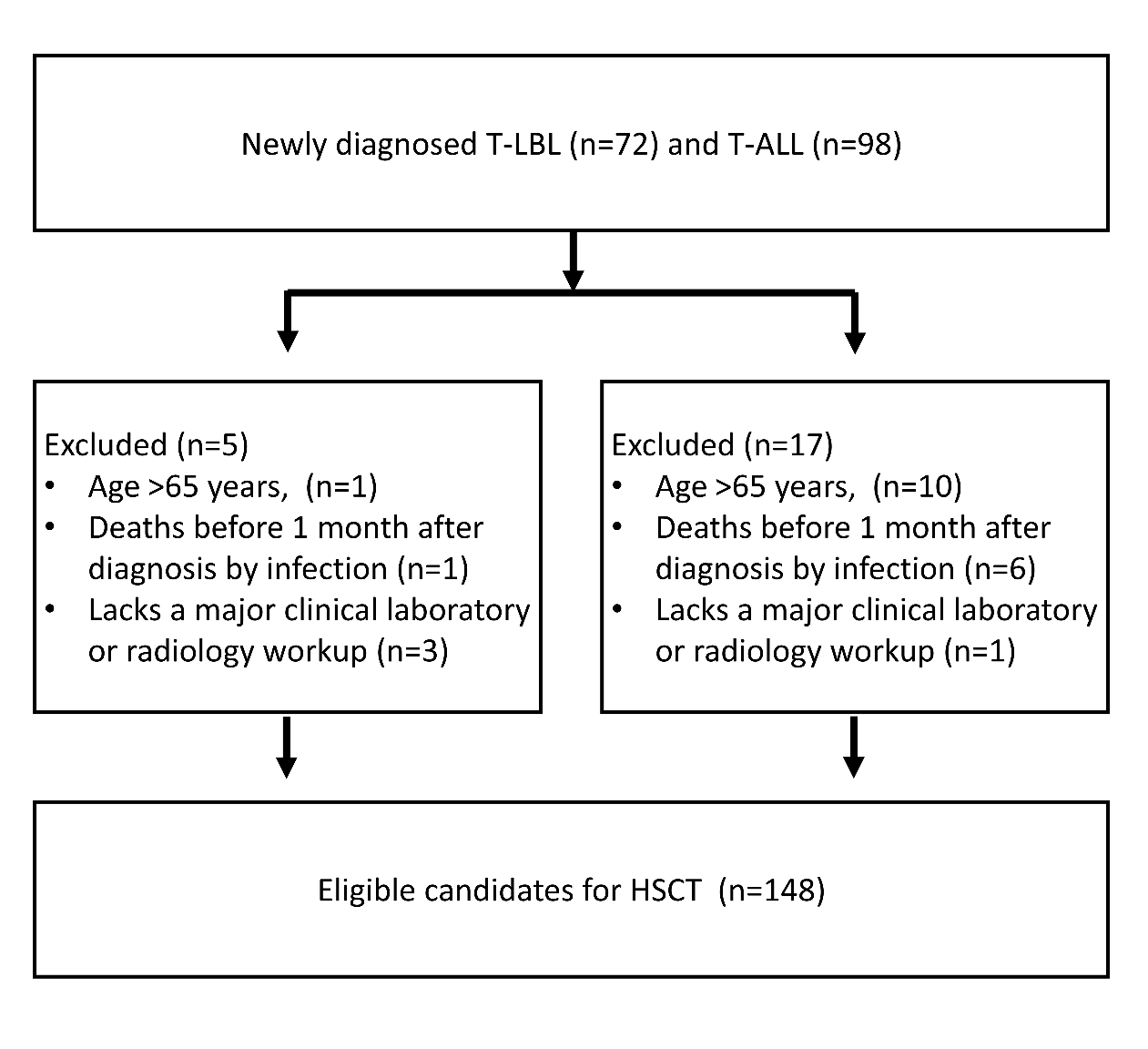
cell acute lymphoblastic leukemia; HSCT, hematopoietic stem cell transplantation.

**
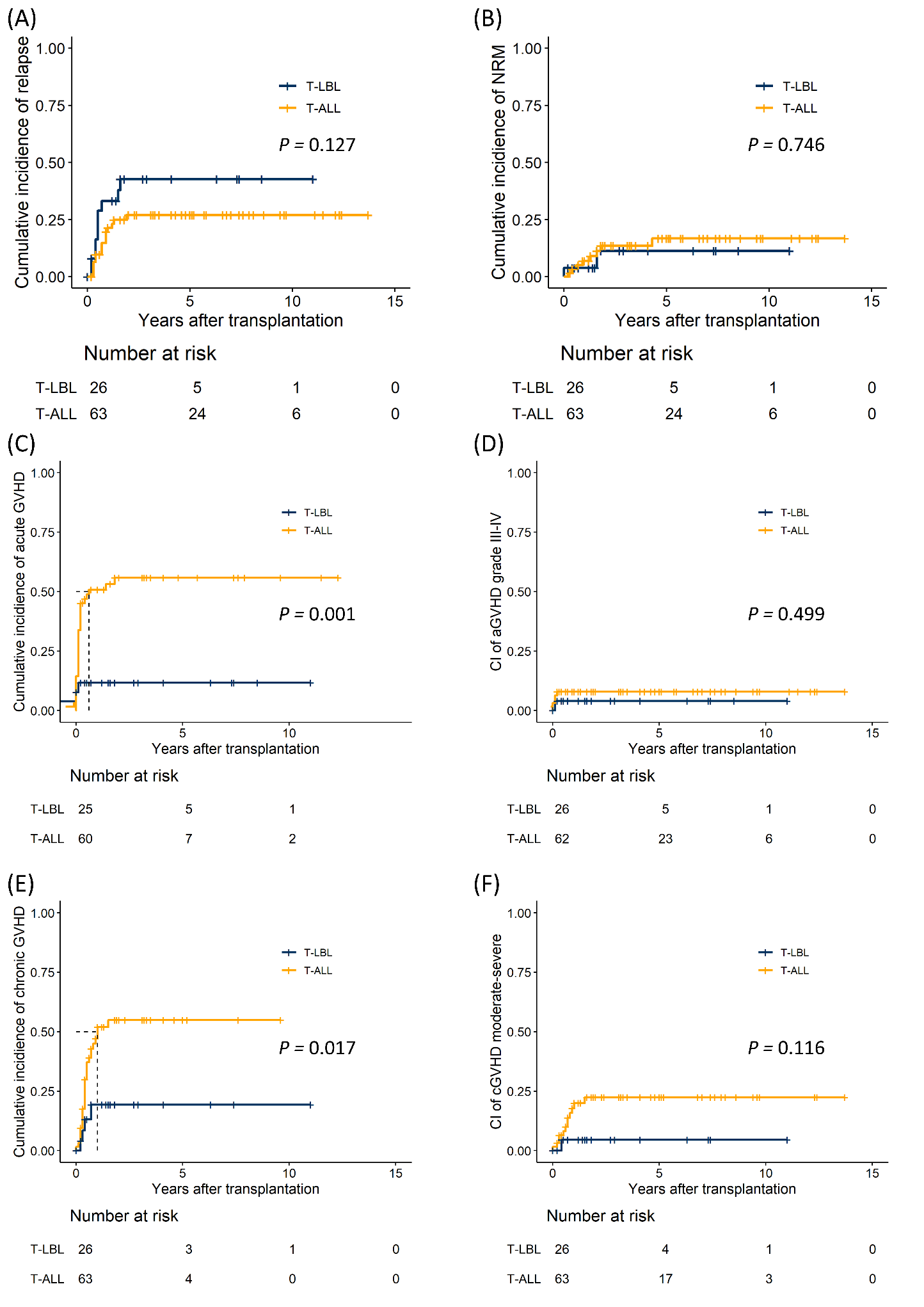
 Figure S2.** Transplantation outcomes for patients with T-LBL and T-ALL. (A) Cumulative incidence of relapse by subtype, (B) cumulative incidence of NRM by subtype, (C) cumulative incidence of acute GVHD by subtype, (D) cumulative incidence of grade III or IV acute GVHD by subtype, (E) cumulative incidence of chronic GVHD by subtype, (F) cumulative incidence of moderate or severe chronic GVHD by subtype.

T-LBL, T-lymphoblastic lymphoma; T-ALL, T-cell acute lymphoblastic leukemia; NRM, non-relapse mortality; GVHD, graft-versus-host disease.
